# Clinical value of sublingual microcirculatory dysfunction for screening for primary aldosteronism in hypertensive patients

**DOI:** 10.3389/fendo.2025.1561503

**Published:** 2025-07-17

**Authors:** Wuhao Wang, Wei Liu, Yi Tang, Fang Sun, Hongbo He, Zhencheng Yan, Qiang Li, Zhiming Zhu

**Affiliations:** ^1^ Department of Hypertension and Endocrinology, Daping Hospital, Army Medical University of PLA, Center for Hypertension and Metabolic Diseases, Chongqing Institute of Hypertension, Chongqing, China; ^2^ Chongqing Institute for Brain and Intelligence, Chongqing, China

**Keywords:** primary aldosteronism, sublingual microcirculation, side-stream dark-field imaging, aldosterone, machine learning

## Abstract

**Background:**

Microcirculation dysfunction commonly occurs in patients with hypertension and diabetes. We aimed to evaluate the changes in sublingual microcirculation among patients with primary aldosteronism (PA), subclinical primary aldosteronism (sPA), essential hypertension (EH), and healthy individuals and aimed to use sublingual microcirculation to screen for PA.

**Methods:**

From January 2023 to January 2024, we consecutively enrolled 191 hypertensive patients (89 EH patients, 51 sPA patients, and 51 PA patients) and 44 healthy individuals. Sublingual microcirculatory images were captured via side-stream dark-field (SDF) microcirculation microscopy, and total and perfused vessel density (TVD and PVD) were calculated. Patient demographic and laboratory data as well as factors influencing microcirculation parameters were assessed.

**Results:**

Compared with healthy individuals (TVD: 13.97 ± 0.62 mm/mm2; PVD: 11.46 ± 0.53 mm/mm2), EH (12.24 ± 0.56; 9.92 ± 0.34), sPA (11.49 ± 0.51; 9.46 ± 0.33) and PA (10.91 ± 0.68; 8.85 ± 0.43) patients exhibited significant microcirculation dysfunction (TVD and PVD). Receiver operating characteristic (ROC) curve analysis revealed that both TVD and the PVD could effectively mirror microcirculation abnormalities in PA patients. We derived a combined evaluation index (CEI) that was composed of the TVD and the PVD for screening PA. By plotting the receiver operating characteristic (ROC) curve, the CEI (AUC: 0.9818 [0.9660, 0.9977]) demonstrated a superior screening effect for PA compared with the aldosterone-to-renin ratio (ARR, AUC: 0.9505 [0.9194, 0.9817]).

**Conclusions:**

Patients with PA had marked microcirculatory dysfunction, which was strongly associated with the ARR. Sublingual microcirculation might be a noninvasive method for the early detection of primary aldosteronism in hypertensive patients.

## Introduction

Primary aldosteronism (PA) is characterized by excessive autonomous secretion of aldosterone from the adrenal cortex. It is the most common cause of secondary hypertension, with a prevalence of 5–15% among hypertensive individuals (1–3). Compared with patients with essential hypertension (EH), PA patients have an increased risk of cardiovascular events such as stroke, myocardial infarction, arrhythmias, and chronic kidney disease (2, 4, 5). In addition to hypertension itself, elevated levels of aldosterone further contribute to target organ damage and increase the risk of cardiovascular events (6, 7). Microvascular abnormalities play an important role in the pathogenesis of target organ damage in hypertension. Structural and functional changes in the microvasculature can lead to tissue hypoxia, metabolic imbalance, and organ dysfunction. Therefore, assessing microvascular function in hypertensive patients can provide important clinical information and improve cardiovascular risk stratification (8).

Currently, invasive detection of microcirculation is difficult to implement widely, especially in earlier screening. Noninvasive detection primarily focuses on microcirculation monitoring in critically ill patients (9). Previous studies have indicated that compared with EH patients, PA patients exhibit more severe changes in skin perfusion and microvascular dysfunction (10, 11). Currently, PA screening and diagnosis rely on biochemical testing of aldosterone and the renin ratio, which has several limitations, such as cumbersome procedures and time-consuming patient waiting times. Sublingual microcirculation, which effectively reflects visceral microcirculation, has been extensively utilized in critically ill patients as a guide for treatment (12, 13). Side-stream dark-field (SDF) imaging is a noninvasive technique that enables real-time direct imaging and quantitative analysis of sublingual microvessels (12). Compared with biochemical tests, this functional test is characterized by its ease of operation, high efficiency, and noninvasiveness. Our previous work demonstrated that SDF imaging effectively detected microvascular abnormalities in diabetic nephropathy (14). Hence, this study aims to evaluate the differences in sublingual microcirculation between healthy individuals and hypertensive and PA patients via SDF imaging and analyze the factors that contribute to these abnormalities. To further explore the early diagnostic value of microcirculatory damage in PA, we also included subclinical primary aldosteronism (sPA) patients in the analysis (15).

## Methods

### Study design and participants

This was a cross-sectional study conducted at Daping Hospital, Army Medical University, Chongqing, China. Between January 2023 and January 2024, a total of 235 subjects were enrolled in the study and classified into four groups: the control group (n=44), the EH group (n=89), the sPA group (n=51), and the PA group (n=51). Except for the healthy control group(n=44), all other patients were recruited from the PA screening population and underwent saline infusion diagnostic tests. In accordance with guideline recommendations (3), drugs that interfere with the RAAS were discontinued for at least 2–4 weeks prior to both the screening and confirmation tests. PA was diagnosed on the basis of the following criteria (16): ① clinical manifestations of hypertension and/or hypokalemia and ② positive results in at least one confirmatory test (in this study, we selected either the captopril challenge test or the intravenous saline suppression test). For patients with hypertension and spontaneous hypokalemia, when the aldosterone-to-renin ratio (ARR) is positive, the renin level is below the detection limit, and when the plasma aldosterone level is > 20 ng/dl, there is no need for confirmatory testing. For positive ARR screening, after the saline infusion test, aldosterone < 5 ng/dL does not support primary aldosteronism, whereas 5–10 ng/dL is considered the sPA group. EH was diagnosed by office blood pressure in accordance with the guidelines of the European Society of Hypertension and the European Society of Cardiology (17). The exclusion criteria included congestive heart failure, liver cirrhosis, renal failure (estimated glomerular filtration rate (eGFR) < 60 ml/min), arrhythmias, severe obesity (body mass index (BMI) > 35 kg/m²), peripheral arterial disease, and a history or clinical signs of any connective tissue disease. All participants underwent sublingual microcirculation assessment via dark-field imaging, and sublingual microcirculation parameters, including total vessel density (TVD, mm/mm²), perfused vessel density (PVD, mm/mm²), and the proportion of perfused vessels (PPV, %), were measured. Demographic characteristics and laboratory data were collected. The study protocol followed the principles of the Helsinki Declaration, and informed consent was obtained from all patients before the initiation of any trial-related activities. The study protocol was approved by the ethics committee of Daping Hospital.

### Sublingual microcirculation collection and preliminary analysis of image data

**Figure 1:**
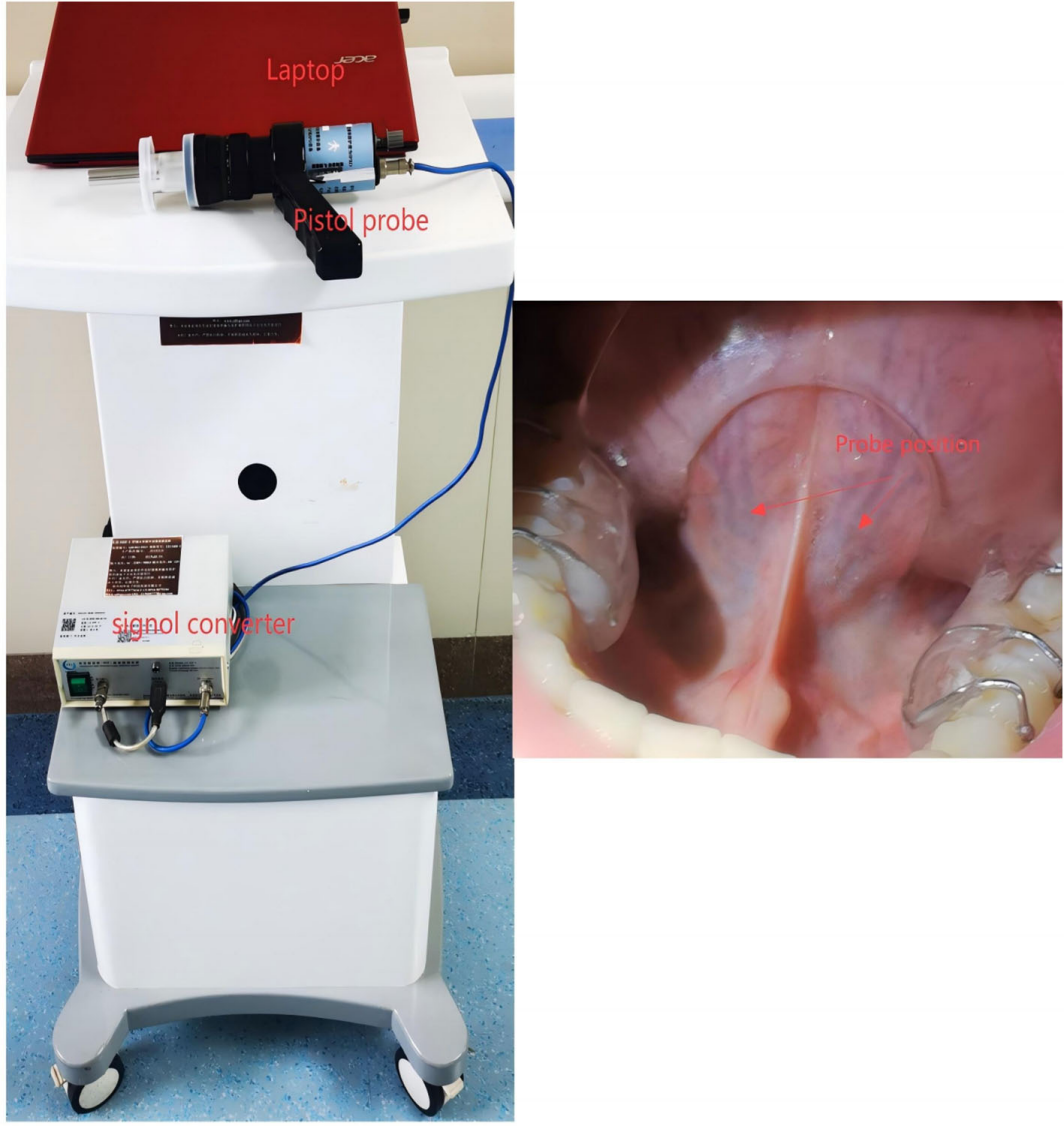
Illustration of sublingual microcirculation image acquisition. The probe head should be placed on either side of the frenulum of the tongue, with either location being optional.

All measurements were conducted in the morning before breakfast. After resting overnight in the ward, patients underwent sublingual microcirculation assessment at approximately 8:00 AM while fasting. Following a brief rinse of the mouth, patients were seated in a temperature-controlled examination room maintained at 25°C and rested for 15 minutes before the sublingual microcirculation examination was performed. All the subjects were instructed not to smoke or consume any food or medications that could affect vascular perfusion within 2 hours before the assessment. The technical details regarding the usage of the equipment have been extensively described in our previous work (14). To enhance the image quality, we contacted the manufacturer to upgrade and improve the equipment. Furthermore, we developed a tongue stabilization device to ensure the relative stability of the examination area and the open-mouth position (**Figure 1**); the arrow indicates the placement point for the probe. We have improved the resolution and recognition system of the probe. The upgraded Chinese- Advanced Vessel Analysis‐Contrast(C-AVA-C) system captured dynamic video of sublingual microcirculation and stabilized the video images for the assessment of blood vessel velocity. It also semiautomatically identifies blood vessel trajectories and generates reports, including the main parameters mentioned in the microcirculation assessment consensus (18, 19). The analysts were blinded to the participants’ information and groups before analyzing and processing the image data. We calculated several sublingual microcirculation parameters, including TVD, PVD and PPV. The measurement methods, significance, and advantages of these parameters in sublingual microcirculation assessment have been described in detail in the consensus (18). Among these parameters, the PVD is considered the gold standard for preclinical research, as it affects capillary distance (diffusive capacity) and red blood cell velocity (convective capacity) (18).

### Laboratory examinations and anthropometric parameter measurements

From the medical record system, we collected subject information, which included name, sex, age, smoking history, drinking history, history of hypertension, comorbidities, plasma aldosterone concentration (PAC), aldosterone/renin ratio (ARR), fasting plasma glucose (FPG), glycated hemoglobin (HbA1c), the urine microalbumin creatinine ratio (UACR), total cholesterol (TC), triglycerides (TG), high-density lipoprotein cholesterol (HDL-c), low-density lipoprotein cholesterol (LDL-c), alanine aminotransferase (ALT), aspartate aminotransferase (AST), serum creatinine (Scr), and the estimated glomerular filtration rate (eGFR). Anthropometric parameter measurements, including blood pressure, waist circumference, height, and weight, were conducted by trained assessors. BMI is defined as weight (kg) divided by height (meter) squared.

### Machine learning methods for screening and diagnosing PA

The machine-assisted random forest algorithm is an ensemble learning algorithm based on decision trees. It uses the Gini coefficient as the feature splitting point and adopts the bootstrap method to randomly extract multiple sample data from the training set. The sample size of each extraction is consistent with the original training set. Then, basic decision tree modeling is performed on each extracted sample. Furthermore, assuming that there are m features in the training set samples, the best feature is selected for splitting during each split until all the training samples at this node belong to the same category. No pruning is performed on the decision tree to allow it to grow to the maximum extent. Finally, the obtained multiple decision trees are combined into a random forest, which is used to classify or regress the new data. For classification problems, multiple tree classifiers are used to determine the best classification result. In regression problems, the final prediction result is the average of the predicted values of multiple trees. In this study, we employed the random forest algorithm to identify the optimal combination of factors for screening and diagnosing PA and constructed relevant models, which were then subjected to external validation.

### Statistical analysis

The results are presented as the means ± standard deviations, medians with interquartile ranges, or frequencies (proportions). The Shapiro–Wilk test was used to determine the normality of distribution for each variable. The chi-square test was used for between-group comparisons of categorical variables. One-way analysis of variance (ANOVA) with the least significant difference (LSD) multiple comparison *post hoc* test was used for continuous variables with a normal distribution. For nonnormally distributed data, the K-sample Kruskal–Wallis one-way ANOVA nonparametric test was used. The intraclass correlation coefficient (ICC) was used for the consistency test. Spearman nonparametric correlation analysis was performed to evaluate the relationships between microcirculation parameters and relevant indicators. Numerical statistical analysis and graphical representation were conducted via SPSS software version 27.0, OriginPro 2021, and GraphPad Prism 9.3.0.

## Results

### Baseline characteristics

The baseline characteristics of all the subjects are shown in **Table 1**. A total of 235 patients were included in the study, with a mean age of 52.16 ± 9.71 years. There were no statistically significant differences in sex, BMI, Scr, eGFR, or TC, TG, ALT, or AST levels among the groups. As expected, PA patients had higher SBP (152.55 ± 10.18 vs. 145.87 ± 10.29, P<0.5), PAC (22.10 (16.00, 27.60) vs. 9.38 (7.04, 14.00), P<0.001), and ARR (8.12 (3.27, 20.40) vs. 0.71 (0.40, 1.56), P<0.001) than did EH patients. Compared with that in the EH group, the DRC in patients with sPA was significantly lower (2.75 (0.87, 5.29) vs. 14.29 (6.08, 29.11), P<0.001). However, no significant difference in the ARR was observed between the sPA and PA groups.

**Table 1:** Baseline characteristics of the patients in each group.

Characteristic	Control (n=44)	EH (n=89)	sPA (n=51)	PA (n=51)
Male sex, no. (%)	17 (38.64)	43 (48.31)	21 (41.18)	19 (37.25)
Age, year	52.84 ± 8.85	51.11 ± 10.52	54.69 ± 9.88	50.88 ± 8.42
BMI, kg/m^2^	23.84 ± 2.55	25.49 ± 3.21*	25.66 ± 3.43*	24.98 ± 3.13
SBP, mmHg	114.11 ± 9.20	145.87 ± 10.29***	147.57 ± 8.47***	152.55 ± 10.18***^#^
DBP, mmHg	70.36 ± 7.93	88.75 ± 9.38***	87.69 ± 11.19***	93.12 ± 12.18***
UACR, mg/g	12.28 (7.34,14.33)	10.85 (6.64,17.45)	13.58 (9.19,24.04)	21.41 (11.41,42.31)*^###^
Scr, μmol/L	71.00 (55.00,84.90)	64.70 (55.15,78.83)	59.60 (54.50,73.50)	59.00 (49.20,74.80)
eGFR, ml/min	117.45 (108.90,146.52)	125.03 (108.64,142.81)	126.66 (110.55,144.40)	135.82 (102.24,154.02)
TC, mmol/L	4.43 ± 1.00	4.57 ± 1.07	4.65 ± 0.98	4.48 ± 0.81
TG, mmol/L	1.47 ± 0.60	2.06 ± 1.27	2.08 ± 2.35	1.75 ± 1.67
HDL-c, mmol/L	1.26 ± 0.30	1.18 ± 0.29	1.19 ± 0.28	1.20 ± 0.22
LDL-c, mmol/L	2.78 ± 0.82	2.79 ± 0.72	2.77 ± 0.64	2.71 ± 0.56
ALT, U/L	19.99 ± 10.14	26.10 ± 20.42	22.23 ± 11.92	21.77 ± 13.63
AST, U/L	22.00 ± 6.56	23.16 ± 9.74	22.86 ± 8.29	22.66 ± 9.29
PAC, ng/dl	10.03 (7.57,13.61)	9.38 (7.04,14.00)	12.50 (10.60,14.40)^#^	22.10 (16.00,27.60)***^###^
DRC, mU/L	10.74 (7.33,17.59)	14.29 (6.08,29.11)	2.49 (1.55,3.99)***^###^	2.75 (0.87,5.29)***^###^
ARR, (ng–dl^-1^)/ (mU–L^-1^)	0.87 (0.69,1.47)	0.71 (0.40,1.56)	4.98 (3.08,8.48)***^###^	8.12 (3.27,20.40)***^###^

Data are mean ± SD, median with interquartile range, or n (%).

SD, standard deviation; BMI, body mass index; SBP, systolic blood pressure; DBP, diastolic blood pressure; UACR, urine microalbumin creatinine ratio; Scr, serum creatinine; eGFR, estimated glomerular filtration rate; TC, total cholesterol; TG, triglycerides; HDL-c, high-density lipoprotein cholesterol; LDL-c, low-density lipoprotein cholesterol; ALT, alanine transaminase; AST, aspartate transaminase; PAC, plasma aldosterone concentration; DRC, direct renin concentration; ARR, aldosterone to renin ratio.

*P < 0.05, ***P < 0.001 vs. control group; ^#^P < 0.05, ^###^P < 0.001 vs. EH group.

### Impairment of sublingual microcirculation in PA patients

**Figure 2:**
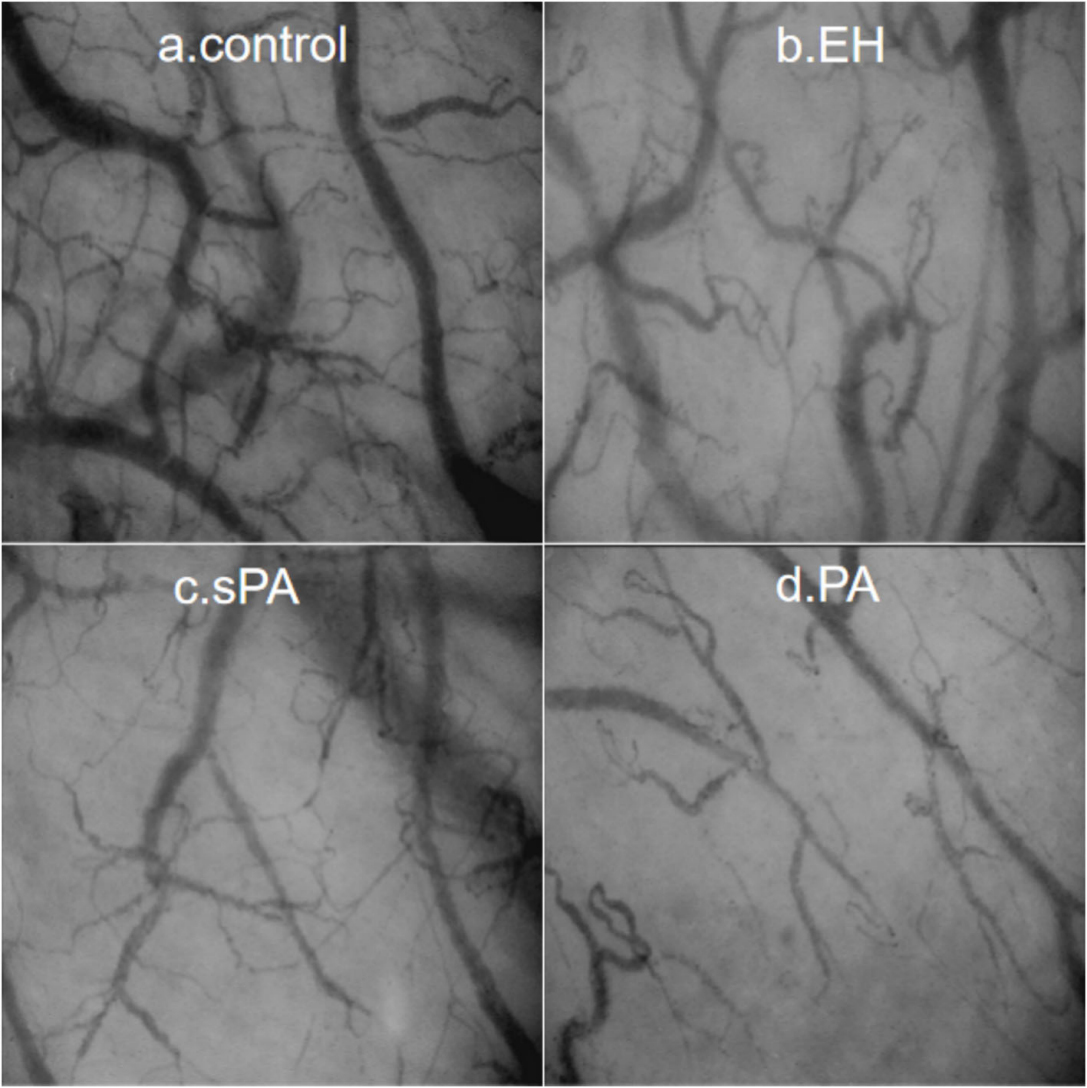
Typical sublingual microcirculation images for each group: **(a)** Control, healthy control group; **(b)** EH, essential hypertension group; **(c)** sPA, subclinical primary aldosteronism group; **(d)** PA, primary aldosteronism group.

Typical images of sublingual microcirculation are shown in **Figure 2**. In the control group, there was no individual red blood cell stasis, the boundary was smooth, and the microcirculation network was rich and uniform. Red blood cell congestion and interrupted vascular boundaries were observed in the EH group, as confirmed by individual red blood cell imaging. In contrast, both the sPA and PA patients presented lower sublingual capillary density, increased vascular tortuosity and stiffness, and a greater proportion of capillary stasis than did the EH patients. Moreover, we calculated three widely recognized microcirculation parameters: total vessel density (TVD), perfused vessel density (PVD), and the proportion of perfused vessels (PPV). The results of the two analysts were consistent (ICC=0.993, P < 0.001).

The microcirculation vessel densities of the control group were significantly greater than those of the other groups (**Table 2**). Compared with those in the EH group, both TVD and PVD were lower in the sPA group, with TVD (11.49 ± 0.51 vs. 12.24 ± 0.56 mm/mm2, P < 0.001) and PVD (9.46 ± 0.33 vs. 9.92 ± 0.34 mm/mm2, P < 0.001) significantly lower. Compared with the EH group, the PA group presented further decreases in TVD (10.91 ± 0.68 vs. 12.24 ± 0.56 mm/mm2, P < 0.001) and PVD (8.85 ± 0.43 vs. 9.92 ± 0.34 mm/mm2, P < 0.001). These findings suggest that sublingual microcirculatory perfusion is more severely impaired in PA patients.

**Table 2:** Comparison of sublingual microcirculation parameters in each group.

Parameters	Control (n=44)	EH (n=89)	sPA (n=51)	PA (n=51)
TVD, mm/mm²	13.97 ± 0.62	12.24 ± 0.56***	11.49 ± 0.51***^###^	10.91 ± 0.68***^###^
PVD, mm/mm²	11.46 ± 0.53	9.92 ± 0.34***	9.46 ± 0.33***^###^	8.85 ± 0.43***^###^
PPV, %	82.05 ± 1.77	81.14 ± 3.41	82.45 ± 3.42^#^	81.24 ± 3.78

TVD, total vessel density; PVD, perfused vessel density; PPV, proportion of perfused vessels.

*P < 0.05, ***P < 0.001 vs. control group; ^#^P < 0.05, ^###^P < 0.001 vs. EH group.

**Figure 3:**
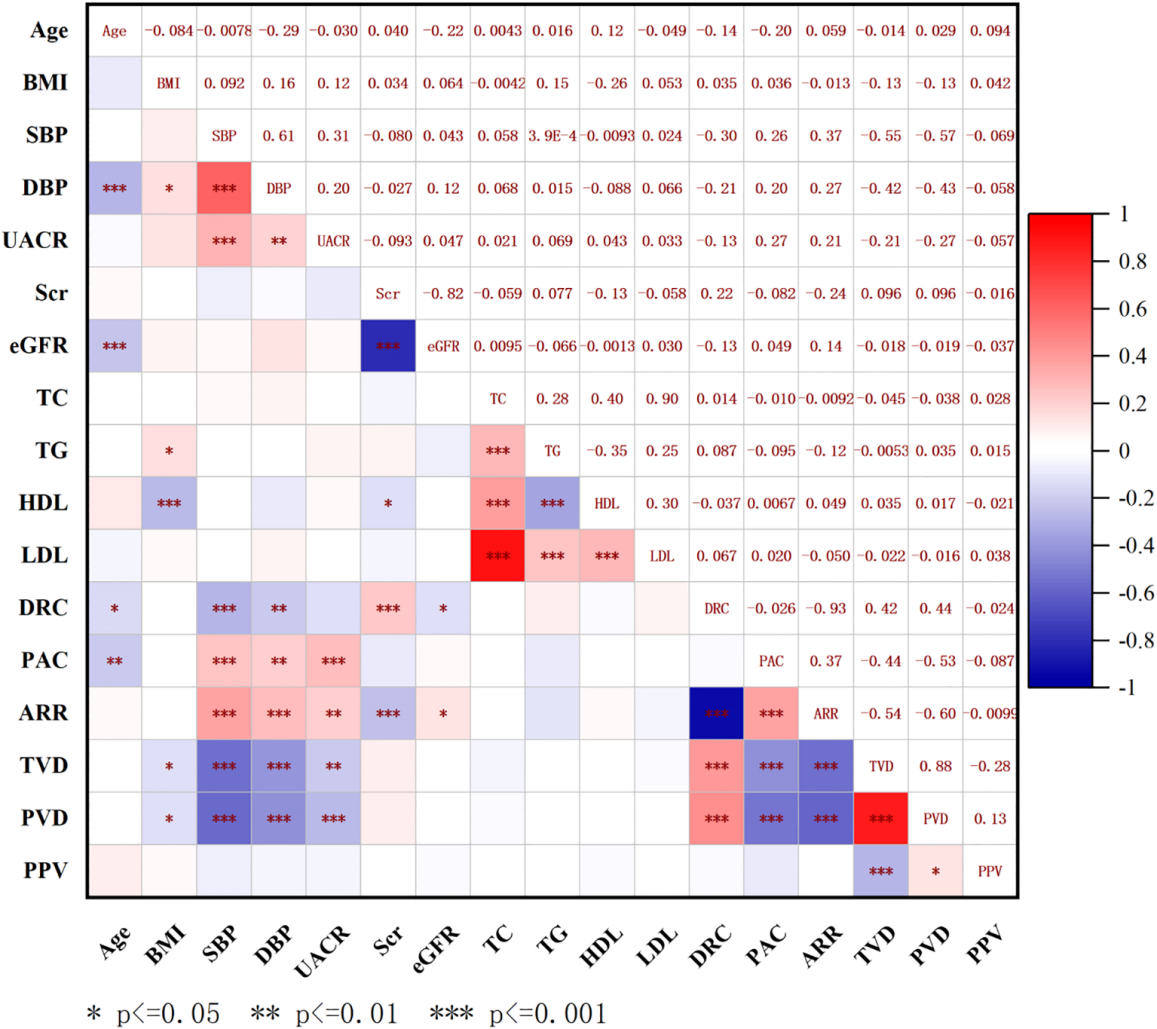
Correlation analysis between sublingual microcirculation parameters and baseline characteristics in all patients. BMI, body mass index; SBP, systolic blood pressure; DBP, diastolic blood pressure; UACR, urine microalbumin creatinine ratio; Scr, serum creatinine; eGFR, estimated glomerular filtration rate; TC, total cholesterol; TG, triglycerides; HDL-c, high-density lipoprotein cholesterol; LDL-c, low-density lipoprotein cholesterol; PAC, plasma aldosterone concentration; DRC, direct renin concentration; ARR, aldosterone to renin ratio; TVD, total vessel density; PVD, perfused vessel density; PPV, proportion of perfused vessels.

To analyze the potential factors associated with the microcirculation parameters, we calculated the correlations between SBP, DBP, BMI, DRC, PAC, ARR, TVD, PVD, and PPV in the PA group. We identified negative correlations between PAC and both TVD and PVD (PAC: TVD, R = -0.440, P < 0.001; PAC: PVD, R = -0.530, P < 0.001) (**Figure 3**). These findings suggest that higher levels of PAC are linked to more severe sublingual microcirculatory damage in PA patients.

### Sublingual microcirculation can be used to screen PA

**Figure 4:**
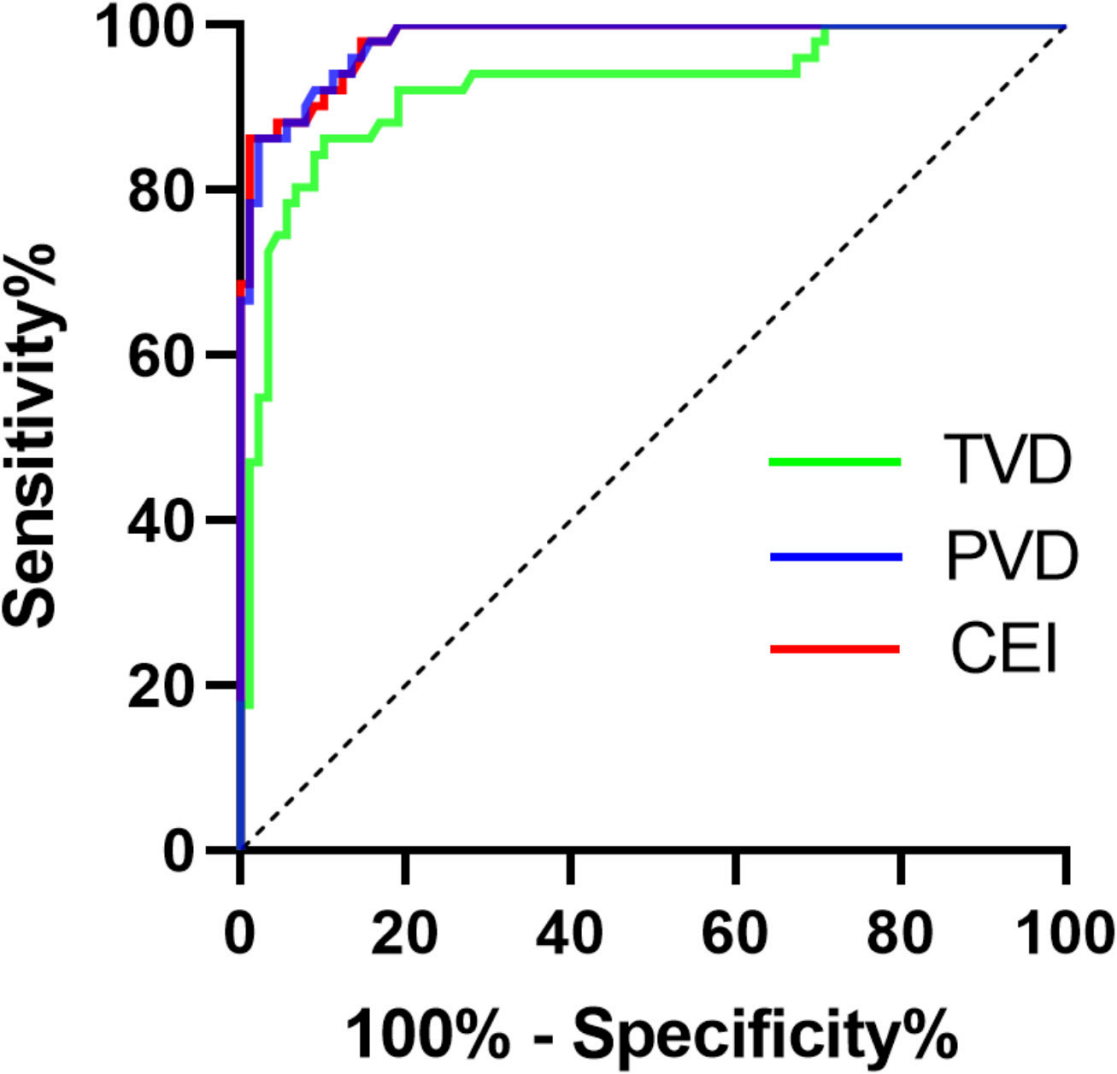
ROC curve analysis of TVD and the PVD for assessing PA. TVD, total vessel density; PVD, perfused vessel density; CEI, combined evaluation index.


Then, we employed TVD and PVD data from both the PA and EH groups to construct ROC curves, which were used to evaluate microcirculatory lesions in PA patients (**Figure 4**). ROC curve analysis revealed that the optimal diagnostic cutoff for TVD was 11.71 mm/mm2, yielding a sensitivity of 86.27%, specificity of 89.89%, and AUC of 0.9223 (95% CI 0.8711–0.9736, P < 0.0001). For the PVD, the optimal diagnostic cutoff was 9.29 mm/mm2, resulting in a sensitivity of 86.27%, a specificity of 97.75%, and an AUC of 0.9812 (95% CI 0.9650–0.9973, P < 0.0001). To enhance the ROC curve performance of the aforementioned microcirculation parameters, we sought to incorporate binary logistic regression analysis to derive a combined evaluation index (CEI). The regression equation was established as follows:

Logit(group)=79.197−1.056×TVD−7.140×PVD

After exclusion of the constant term, we proceed with the following calculations:


CEI=TVD+(7.140×PVD)/1.056

ROC curve analysis revealed that the optimal diagnostic cutoff for the CEI was 85.15 mm/mm2, yielding a sensitivity of 86.27% and specificity of 98.88%. The AUC was 0.9818 (95% CI:0.9660–0.9977, P < 0.0001). Compared with individual TVD or PVD, the CEI exhibited superior evaluation performance. Compared with ARR (AUC: 0.9505 [0.9194, 0.9817]) and confirmatory tests, this method can significantly simplify the inspection and testing procedures.

### Establishment of a composite parameter model via machine learning

**Figure 5:**
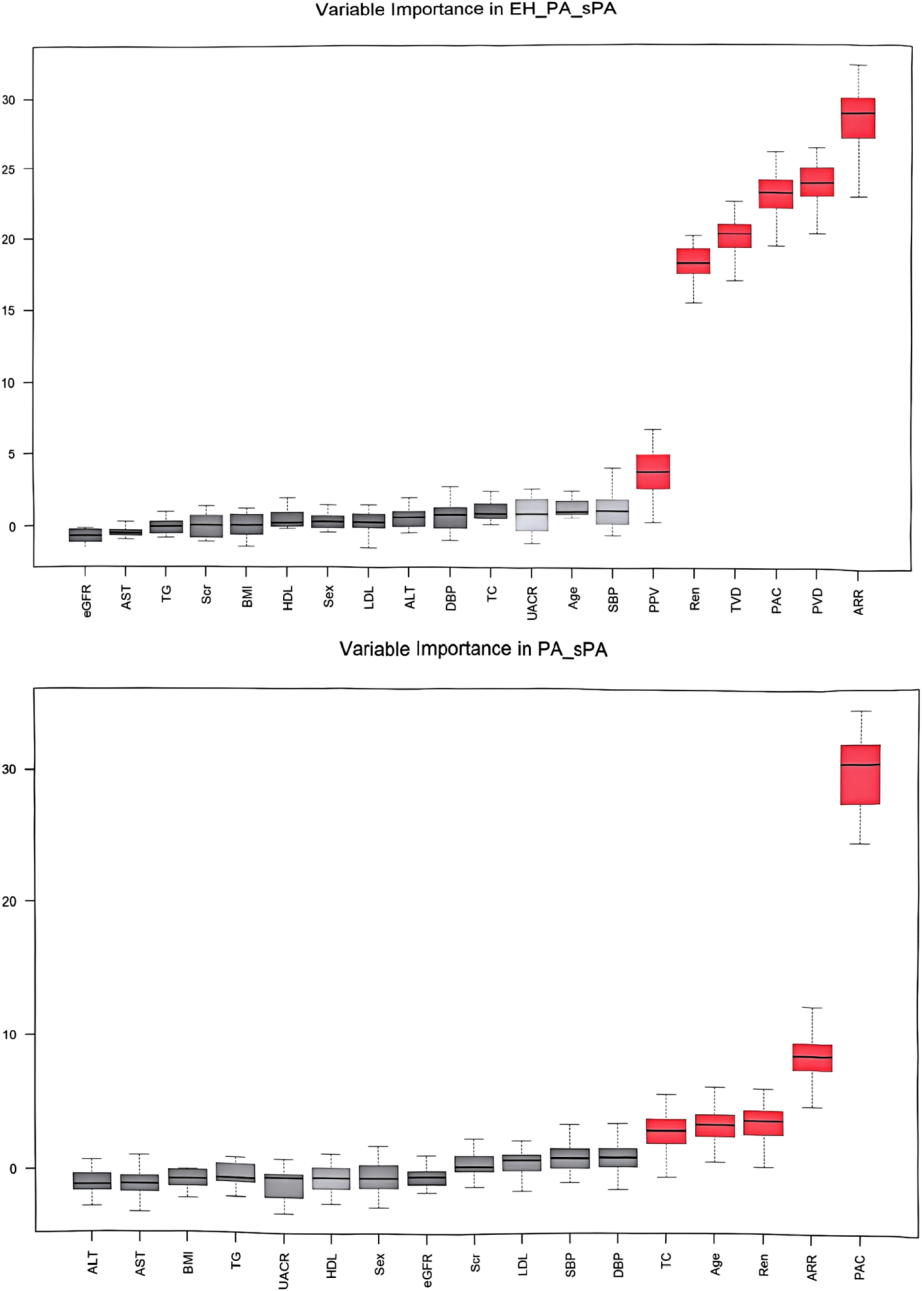
Variable importance in screening and diagnosis models. BMI, body mass index; SBP, systolic blood pressure; DBP, diastolic blood pressure; UACR, urine microalbumin creatinine ratio; Scr, serum creatinine; eGFR, estimated glomerular filtration rate; TC, total cholesterol; TG, triglycerides; HDL-c, high-density lipoprotein cholesterol; LDL-c, low-density lipoprotein cholesterol; ALT, alanine transaminase; AST, aspartate transaminase; PAC, plasma aldosterone concentration; DRC, direct renin concentration; ARR, aldosterone to renin ratio; TVD, total vessel density; PVD, perfused vessel density; PPV, proportion of perfused vessels.

Through the establishment of the ROC curve based on the sublingual microcirculation parameters, we found that these parameters could be applied in the screening of PA. Therefore, we further explored the feasibility of establishing a predictive model for PA screening among hypertensive patients admitted to the hospital. To determine the optimal parameter set for the predictive model, we employed a random forest classifier and utilized a grid search for disease diagnosis classification. The importance of the parameters after screening is shown in **Figure 5**. We finally established prediction model steps for screening sPA and PA in patients with hypertension.

First, the sPA or PA population (EH vs sPA+PA) was screened out:

Logit (sPA & PA) = 0.893 * DRC + 2.827 * PVD + 2.510 * TVD + 0.182 * ARR - 0.499 * PAC.

Furthermore, we calculated the cutoff point for sPA&PA (57.32, AUC = 0.9082 [0.8464, 0.9700], sensitivity=98.04%, specificity=94.38%).

Then, among the suspicious population, the PA cases (sPA vs. PA) were further screened out:

Logit (PA) = -0.195 * DRC - 0.154 *PAC - 0.146 * ARR - 0.183 * TVD + 2.611 * PVD.

The cutoff point for PA was 17.89 (AUC = 0.9082 [0.8464, 0.9700], sensitivity=82.35%, specificity=86.27%).

To validate the effectiveness of both the screening and diagnostic prediction models, we conducted external validation by recruiting 107 patients with hypertension who were admitted to the hospital and underwent sublingual microcirculation testing. We compared the results of the prediction model with the preliminary clinical screening results and performed a kappa consistency test. For the screening experiment, namely, the selection of the sPA or PA population, the kappa value was 0.810, P<0.001. Furthermore, we conducted a consistency test for the diagnostic prediction model to distinguish PA patients from the sPA or PA population, and the kappa value was 0.827 (P<0.001). For both screening and diagnosis, the combined prediction model incorporating sublingual microcirculation indicators, aldosterone, and renin hematological examination indicators has good applicability and effectiveness.

## Discussion

Our findings demonstrate a significant impairment in microcirculation among both sPA and PA patients compared with EH patients. This impairment is evident through the decreased microcirculatory parameters of TVD and PVD. Importantly, we observed a strong association between aldosterone levels and both TVD and PVD in PA patients. Additionally, TVD and PVD exhibited high sensitivity in detecting microcirculation impairment in PA patients. To further enhance the analysis indicators, we derived a combined evaluation index that integrates TVD and PVD. This combined functional index is characterized by its simplicity and efficiency, which holds promise for simplifying traditional screening and diagnostic tests for PA.

Excess aldosterone can lead to sodium retention, potassium loss, increased blood volume, and secondary hypertension (20). The early stages of hypertension are closely related to microvascular damage, including structural and functional changes (21). However, microvascular damage in PA is not solely attributed to increased blood pressure; in fact, aldosterone itself has other nonclassical effects, including increasing the expression of growth factors that control tissue growth and mediating genes involved in inflammation, leading to microvascular damage in the heart, vascular system, and kidneys (22, 23). The harmful effects of this action are further amplified in PA, as excessive aldosterone significantly increases oxidative stress, mediates inflammation, and promotes endothelial dysfunction and fibrosis (24).

Microvessels can be considered windows for observing cardiovascular and metabolic diseases (25–27). The evaluation of the microvascular phenotype can provide clinically relevant information before and during treatment, greatly improving cardiovascular risk stratification (8). Noninvasive, clinically reliable, and easy-to-perform techniques such as SDF imaging and incident dark field (IDF) imaging are currently hot research topics (9). The sublingual microcirculation is rich in blood vessels and can reflect the condition of the visceral microcirculation. It has been widely used in the monitoring and guidance of treatment in critically ill patients (28–30). SDF technology combined with a handheld video microscope (HVM) can measure microvessel diameter, total vessel density, and perfused vessel density. The microcirculation in the sublingual area is often studied via HVM because the vascular changes detected in this area are closely associated with the risk of cardiovascular and noncardiovascular morbidity and mortality in patients (31). The European Society of Intensive Care Medicine also considers sublingual microcirculation as a relevant parameter for critically ill patients (17). Additionally, the use of nitroglycerin (32) or acetylcholine (33) in the sublingual area can provide the possibility to assess the microcirculatory reserve. Previous studies have also utilized SDF imaging technology to detect changes in sublingual blood flow perfusion in patients with hypertension and diabetes (14, 34).

ARR has become the standard screening method for primary aldosteronism (PA) (3). However, this method has the drawbacks of low screening rates and insufficient accuracy. In recent years, multiple studies have supported active follow-up or early intervention for subclinical PA (35–41). Currently, biochemical tests may be replaced by more convenient and efficient functional tests. Our sublingual microcirculation tests revealed a significantly greater decrease in total vessel density and perfused vessel density in the PA and sPA groups than in the EH group. The composite index of sublingual microcirculation demonstrates good applicability in the diagnosis of PA.

This study has several limitations. First, this study is a single-center, cross-sectional observational study. As a diagnostic test, it is unable to establish a causal relationship between sublingual microcirculatory dysfunction and PA. Although the patients with PA were recruited from a single center, they were sourced from the Chongqing Endocrine Hypertension Collaborative Team (42–46). Second, this is a pilot study with a relatively small sample size, which may not fully capture the diversity of the PA population. Third, the imaging analysis currently employs semiautomatic identification of blood vessel trajectories and reports generation, which may introduce biases in the interpretation of our findings. We had implemented several measures to minimize potential biases and improve the reliability of image analysis. Future work will focus on exploring the integration of automated image analysis techniques to further improve the accuracy and reliability of our image analysis.

In summary, we evaluated sublingual microcirculation changes in PA patients via SDF imaging technology. We reported that PA and sPA patients presented a more pronounced decrease in vessel density than EH patients. We found higher levels of plasma aldosterone concentration are linked to more severe sublingual microcirculatory damage in PA patients. Through future validation in a larger population, screening sublingual microcirculation via SDF imaging technology is expected to become a noninvasive detection method for the early screening of PA.

## Data Availability

The original contributions presented in the study are included in the article/supplementary material. Further inquiries can be directed to the corresponding authors.
